# Propolis supplementation improved productivity, oxidative status, and immune response of Barki ewes and lambs

**DOI:** 10.14202/vetworld.2019.834-843

**Published:** 2019-06-18

**Authors:** Hesham Attia Shedeed, Bahaa Farrag, Eman Ali Elwakeel, Ibrahim Samir Abd El-Hamid, Muhammed Ahmed-Hilmy El-Rayes

**Affiliations:** 1 Animal and Poultry Production Division, Desert Research Center, Ministry of Agriculture and Land Reclamation, Egypt; 2 Department of Animal and Fish Production, Faculty of Agriculture, Alexandria University, Alexandria 21545, Egypt

**Keywords:** antioxidants, Chinese propolis, immunoglobulin, productivity, sheep, thermorespiratory responses

## Abstract

**Aim:**

The present study was conducted to study the effect of propolis administration on bio-hematological parameters, antioxidant enzyme activities, and productivity of Barki ewes during late pregnancy and lactation under the arid conditions.

**Materials and Methods:**

Twenty-five pregnant Barki ewes were fed the basal diet (n=12, control) and the basal diet plus propolis (5 g/kg diet, n=13) for 1 month before parturition and continued 2 months after parturition. Milk yield and milk composition, hematological constituents, antioxidant enzyme activities, thyroid hormones, and lambs birth and weaning weights, and antioxidants were determined.

**Results:**

Significant (p<0.05) increase in white blood cells in the propolis group compared to control was observed. Mean corpuscular hemoglobin (Hb) (MCH) and corpuscular Hb (MCH concentration %) were decreased (p<0.05) in propolis compared to control group. Milk yield was increased (p<0.05) in the propolis group compared with control and continued to increase with the advancement of lactation. Milk fat and milk total solids increased (p<0.05) in the propolis group than the control. Plasma immunoglobulin A (IgA) was increased (p<0.05) in propolis compared to control with no effect in IgM and IgG. Superoxide dismutase, hydrogen peroxide (HP), and nitric oxide were decreased (p<0.01) in the propolis group compared to control. Weaning weight for lambs born to ewes fed propolis was increased (p<0.05) at week 8 after birth compared with control lambs. Malondialdehyde and HP activities were decreased (p<0.01) in lambs born to propolis ewes compared to control.

**Conclusion:**

Crude Chinese propolis (5 g/d) supplementation improved milk yield, milk composition, and the antioxidant enzymes in Barki ewes and immune functions, growth performance and antioxidant status in their lambs under arid conditions.

## Introduction

Sheep meat is considered the second source of red meat after beef and buffalo and contributes 6% of the total red meat production in Egypt [1]. Barki breed is characterized by its high meat quality and adaptability to poor pasture and harsh desert conditions compared with other local breeds [2]. It is believed that Barki sheep originated from Barkah region at the Republic of Libya and are raised on the west-coast line of the Mediterranean and their population in Egypt is 470.000 heads [3]. The productivity of Barki sheep was reported to be low due to several factors including environment, health conditions, and scarcity of feed which leads to higher oxidative stress and hyperketonemia during late pregnancy when fetal requirements are high [4]. Therefore, studies have been conducted experimenting with the use of natural additives to improve animal health in arid and semi-arid zones [5]. Others have found ways to increase growth rate and improve animal’s welfare without increasing feed intake by offering lambs free choice feeding rather than total mixed ration feeding [6]. The World Health Organization banned the use of non-natural sources of diet such as synthetic compounds (e.g., antibiotic) because they pose serious impacts on the health of animals and humans [7].

Propolis is a natural resinous product that honeybees collect from several plants and mix it with beeswax and salivary enzymes [8]. Recent studies have used it to improve animal productivity due to its biological and pharmacological properties [5]. The chemical composition and color of propolis (brown, green, and red) differ from area to area depending on the vegetation of the surrounding zone [9]. Several studies concluded that the propolis has many vital functions such as antimicrobial, anti-inflammatory activities [10], antioxidant [11], immuno-modulatory [12], anti-tumor [13], antiviral [14], and antibacterial [15].

The present study was conducted to study the effect of administration of Chinese propolis on some bio-hematological parameters, antioxidant enzyme activities, and some productivity traits of Barki ewes during late pregnancy and lactation under the arid conditions of Egypt.

## Materials and Methods

### Ethical approval

Experiments were carried out in accordance with the guidelines laid down by the Institute of Animal Ethics Committee for the use of animals (2010/63/EU of the European Parliament and of the Council of September 22, 2010).

### Study area

This study was carried out at Tegzerty Research Station, Siwa Oasis, Western Desert of Egypt located 65 km of Libyan borders (Latitude: 29° 06” 29° 24” N and Longitude: 25° 16” 26° 12” E). This research station belongs to Desert Research Center, Ministry of Agriculture and Land Reclamation, Egypt.

### Animals, management, and experimental design

**Figure-1:**
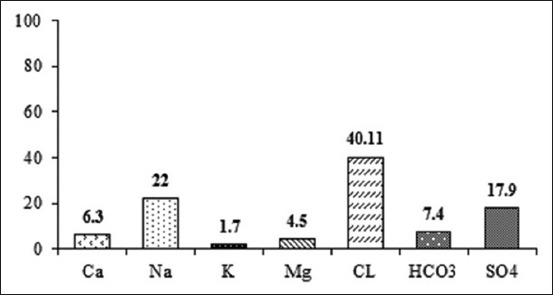
Chemical analysis of drinking water in Siwa Oasis.

Twenty-five pregnant multiparous Barki ewes, 3-5 years old with an average body weight of 48±0.23 kg (mean ± standard deviation) were used in this study. The study was conducted from August 2016 to November 2016. The ewes were housed in freely ventilated semi-closed pens throughout the experimental period. Ewes were fed a concentrated mixture according to their body weight requirements [16]. All animals were given berseem hay as roughage *ad libitum*. Each animal received 1 kg/h/day of pelleted concentrate mixture that contained 65% total digestible nutrients and 14% crude protein (Table-1). Fresh underground water was presented twice daily at 08:00 am and 03:00 pm. The chemical analysis of water (Figure-1) was carried out according to the American Society for Testing and Materials [17].

**Table-1:** Ingredients and chemical composition of concentrate mixture and berseem hay.

Ingredients of concentrate mixture		g/kg*
Yellow corn		250
Cottonseed meal		169.7
Wheat bran		300
Sunflower meal		250
NaCl		10
Limestone		20
Trace minerals**		0.3

**Chemical composition (g/kg)**	**Concentrate mixture**	**Berseem hay**

Organic matter	940	858
Ash	060	142
Crude protein	148	154
Ether extract	055	013
Neutral detergent fiber	534	486
Acid detergent fiber	369	352
Hemicellulose	165	134

* ME=843±25.2 kJ/kg^0.75^,

** trace minerals ontained (g/kg): Manganese sulphate 12.58, zinc sulfate 9.3, copper sulfate 3.2, ferrous sulfate 16.67 calcium iodate 0.081, sodium selenite 0.4, magnesium oxide 9.4, cobalt sulfate 0.2, sodium chloride added to kg. (Dyno Vet Company, Alexandria, Egypt)

Twenty-five multiparous, late pregnant (120 days) Barki ewes were divided into two groups: The first group: Control (n=12) received the basal diet only and the second group (n=13) received the basal diet plus (5 g/kg diet) of Chinese propolis powder (Manufacturer, Trading Company, Henan, China), the chemical composition was determined by gas chromatography-mass [18] at our laboratory. Propolis additive was mixed with concentrate diet and fed to ewes for 3 months. Administration of propolis started 1 month before parturition (late pregnancy, 120 days) and continued for 2 months after parturition (early weaning).

### Meteorological data

**Figure-2:**
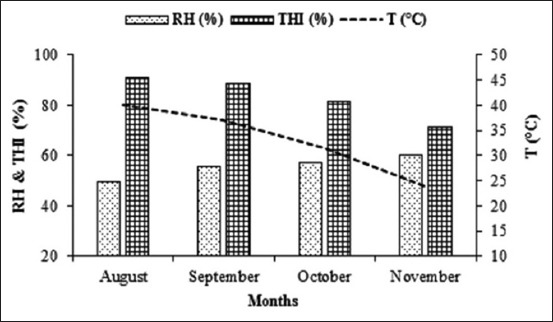
Changes in mean ambient temperature (°C) and relative humidity (%) and temperature-humidity index percentage throughout the experimental period.

Meteorological data including ambient temperature (°C), relative humidity (RH, %) were recorded using hygro-thermometer. The mean temperature-humidity index (THI) was calculated according to equation: THI= (0.8×AT°C)+[(RH/100)× (AT°C–14.4)]+46.4 [19]. The mean values of these parameters are presented in Figure-2.

### Blood sampling and hematological analysis

Blood samples were collected from the jugular vein in plasma vacutainer tubes (K_3_ EDTA) from all ewes. Collection of samples was carried out at day-30 (before the start of Chinese Propolis administration, day-15 (late pregnancy stage), and 0, 15, and 30 days after parturition. A part of the samples was withheld as whole blood to determine hematological parameters; plasma was then harvested after centrifugation at 5000 g for 10 min and then stored at −20°C for later analysis. Blood samples of lambs were collected from the jugular vein in plasma vacutainer tubes (K_3_ EDTA) monthly after parturition and continued until early waning (60 days after parturition).

#### Hematological parameters

Hemoglobin (Hb) concentration was determined using colorimetric kits (Vitro Scient, Egypt). Packed cell volume percent (PCV, %) was estimated by the hematocrit tubes, the number of red blood cells (RBC’s, 10^6^/mm^3^) and total count of white blood cells (WBC’s, 10^3^/mm^3^) were counted by Thom’s hemocytometer slide. Mean corpuscular volume (MCV, µm^3^) was calculated according to equation MCV, µm^3^=PCV (%)/RBC’s (10^6^/mm^3^)×10. Mean corpuscular Hb (MCH, pg) was calculated according to equation MCH, pg = Hb (g/dl)/RBC’s (10^6^/mm^3^)×10. MCH concentration (MCHC, %) was calculated according to equation MCHC, %=Hb (g/dl)/PCV (%)×100.

#### Plasma immunoglobulin (Ig)

Plasma Ig including (IgG, IgA, and IgM) was measured using ELISA kits purchased from Abbott Park, IL 60064, USA.

#### Plasma antioxidant enzyme activities

Antioxidants enzyme activities of superoxide dismutase (SOD), malondialdehyde (MDA), hydrogen peroxide (HP), and nitric oxide (NO) were assayed calorimetrically using commercial kits (Biodiagnostic Research, Egypt). Triiodothyronine (T_3_) and thyroxine (T_4_) hormones were analyzed using ELISA kits (Atlas Medical, London). The intra- and inter-assay coefficient of variations were 8.83 and 8.86%, respectively.

### Milk yield and milk composition

Milk yield was recorded biweekly for individual ewes starting from week 2 after parturition until week 8 of lactation period using hand milking technique. Milk samples (50 ml) were taken biweekly in plastic containers to determine fat, protein, lactose, total solid (TS), solid not fat (SNF), and ash content using Milkoscan (Bentley - Belgium).

### Body weight of lambs

Body weight of newly born lambs (birth weight), biweekly weights, and weaning weights at week 8 after parturition was recorded using the digital balance.

### Thermo-cardiorespiratory parameters of lambs

Thermo-cardiorespiratory parameters were measured at days 30 and 60 after parturition at 08:00 am and 02:00 pm. Rectal temperature (RT, °C) was measured using a clinical thermometer. Skin temperature (ST, °C) and coat temperature (CT, °C) were measured using IR thermometer laser Class 2 (Cooper - ONDA 630-670 nm, USA). Respiration rate (RR, rpm) was measured by counting flank movements per minute. Heart rate (HR, bpm) was measured as beats per minute using a clinical stethoscope.

### Statistical analysis

Data of ewes and newborn lambs’ body weight, for 8 weeks after birth, antioxidant enzymes were analyzed by General Linear Model (GLM) procedure [20] using the following model: Y_ijk_=µ+G_i_+A(G)_ik_+Day_j_+G*Day_ij_+e_ijk._Y_ijk_=observations. µ=overall means. G_i_=effect of i^th^ group (i: 1-2). A (G)_ik_=the repeated k^th^ animals within i^th^ treatment day_j_=effect of j^th^ day (j: 0-5). G*Day_ij_=interaction between groups and days. e_ijk_=experimental error. Data for newborn lambs’ thermo-cardiorespiratory parameters (RT, ST, CT, RR, and HR) for 30 and 60 days after birth were analyzed by the GLM procedure using the following model:


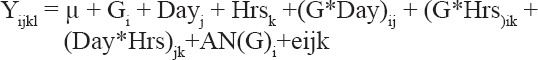


Yijkl=observation. µ=overall means. G_i_=effect of i^th^ group (i: 1-2), Day_j_=effect of j^th^ day (j: 0-2). Hrs_k_=effect of k^th^ hours (j: 0-2). G_i_*Day_j_=effect of the interaction of groups × days. AN (G)_i_=error 1. G_i_*Hrs_k_=effect of the interaction groups × hours.

e_ijk_=experimental error. Duncan’s multiple range tests were used to separate means. Significance was declared at p<0.05 and tendencies at 0.05≤p≤0.10.

## Results

### Changes in Igs concentrations

Changes in plasma Igs concentrations in pregnant and lactating Barki ewes are presented in Table-2. No treatment × time interaction was observed on plasma Igs. However, overall mean of IgG (IgG, mg/mL) tended to slightly decrease (p=0.08) in propolis group (2.43 mg/mL) compared with control (2.46 mg/mL). Overall mean IgM (IgM, mg/mL) concentration was increased (p<0.05) in propolis group (0.55 mg/mL) compared to control (0.45 mg/mL). Further, overall mean of IgA concentrations was increased (p<0.05) in propolis group (0.34 mg/mL) compared to control (0.28 mg/mL).

**Table-2:** Changes in plasma Igs (IgG, IgM, and IgA) in Barki ewes fed a basic diet or a diet supplemented with propolis during late pregnancy to early lactation.

Item	Treat.	Days					Overall	SEM	p-value		
	
		−30	−15	0	15	30			T	D	T×D
IgG, (mg/mL)	Control	2.49	2.46	2.45	2.45	2.45	2.46	0.02	0.08	0.51	0.97
	Propolis	2.45	2.42	2.43	2.43	2.41	2.43	0.02			
IgM, (mg/mL)	Control	0.36	0.43	0.42	0.52	0.51	0.45^B^	0.09	0.05	0.53	0.87
	Propolis	0.57	0.44	0.50	0.58	0.65	0.55^A^	0.01			
IgA, (mg/mL)	Control	0.26	0.26	0.30	0.29	0.32	0.28^B^	0.01	0.05	0.22	0.39
	Propolis	0.35	0.32	0.31	0.35	0.36	0.34^A^	0.02			

A-B Values within the same column with different letters differ significantly. Days −30 and −15: Days of late pregnancy, Day 0: Day of parturition. Days 15 and 30: Days of early lactation. IgG=Immunoglobulin G, IgA=Immunoglobulin A, IgM=Immunoglobulin M, SEM=Standard error of the mean

### Changes in some antioxidant activities

Changes in plasma MDA (nM/mL), SOD (U/mL), HP (mM/mL), and NO (µM/L) antioxidant enzyme concentrations in Barki ewes are presented in Table-3. No treatment × time interaction was observed on four plasma antioxidant enzyme activities. Treatment with propolis decreased (p<0.05) significantly the overall plasma concentrations of MDA (5.82 vs. 6.52 nM/mL), SOD (3.25 vs. 6.07 U/mL), HP (0.42 vs. 0.46 mM/mL), and NO (18.52 vs. 41.88 µM/L) compared to the control, respectively.

**Table-3:** Changes in plasma antioxidant enzyme activities in Barki ewes fed a basic diet or a diet supplemented with propolis during late pregnancy and early lactation.

Items	Treat.	Days					Overall	SEM	p-value		
	
		−30	−15	0	15	30			T	D	T×D
MDA (nM/mL)	Control	6.19	7.64	7.14	6.14	5.51	6.52^A^	0.59	0.05	0.25	0.54
	Propolis	6.04	5.96	5.84	5.74	5.52	5.82^B^	0.59			
SOD (U/mL)	Control	6.67	9.36	6.13	3.17	5.03	6.07^A^	1.99	0.05	0.01	0.93
	Propolis	5.19	6.65	1.87	0.89	1.68	3.25^B^	2.02			
HP (mM/mL)	Control	0.48	0.48	0.45	0.46	0.45	0.46^A^	0.01	0.05	0.63	0.87
	Propolis	0.43	0.42	0.43	0.42	0.41	0.42^B^	0.02			
NO (mM/L)	Control	44.67	40.7	39.5	39.2	45.4	41.88^A^	0.79	0.05	0.25	0.44
	Propolis	13.12	16.3	15.8	18.2	29.2	18.52^B^	0.79			

A-B Values within the same column with different letters differ significantly (p<0.05). Days −30 and−15: Days of late pregnancy, day 0: Day of parturition. Days 15 and 30: Days of early lactation. MDA=Malondialdehyde, SOD=Superoxide dismutase, HP=Horse radish peroxidase, NO=Nitric oxide, SEM=Standard error of the mean

### Changes in plasma thyroid hormones concentrations

Changes in plasma T_3_ and T_4_ concentrations in Barki ewes are shown in Table-4. No treatment × time interaction was observed on plasma T_3_ concentrations. The differences in overall means plasma T_3_ (T_3_, ng/mL) concentrations between propolis and control groups (1.48 vs. 1.45 ng/ml, respectively) were not significant (p=0.54). The same trend was observed in plasma T_4_ concentrations where no significant (p=0.91) differences were detected in overall means of plasma T_4_ (ng/mL) concentrations between propolis and control groups (7.08 vs. 7.13 ng/mL, respectively). Plasma T_3_ and T_4_ concentrations in both control and propolis groups decreased before parturition then rebound back at days 15 and 30 after parturition (Table-4).

**Table-4:** Changes in plasma triiodothyronine (T_3_) and thyroxin (T_4_) concentrations in Barki ewes fed a basic diet or supplemented with propolis during late pregnancy and early lactation period.

Items	Treat.	Days					Overall	SEM	p-value		
	
		−30	−15	0	15	30			T	D	T×D
T3, (ng/mL)	Control	1.51	1.56	1.35	1.47	1.53	1.48	0.08	0.54	0.01	0.37
	Propolis	1.54	1.39	1.23	1.42	1.67	1.45	0.08			
T4, (ng/mL)	Control	7.66	5.44	5.15	8.51	8.89	7.13	0.66	0.91	0.01	0.83
	Propolis	7.70	6.16	5.34	8.05	8.16	7.08	0.66			

T_3_=Triiodothyronine, T_4_=Thyroxin, SEM=Standard error of the mean

### Changes in hematological parameters

**Figure-3:**
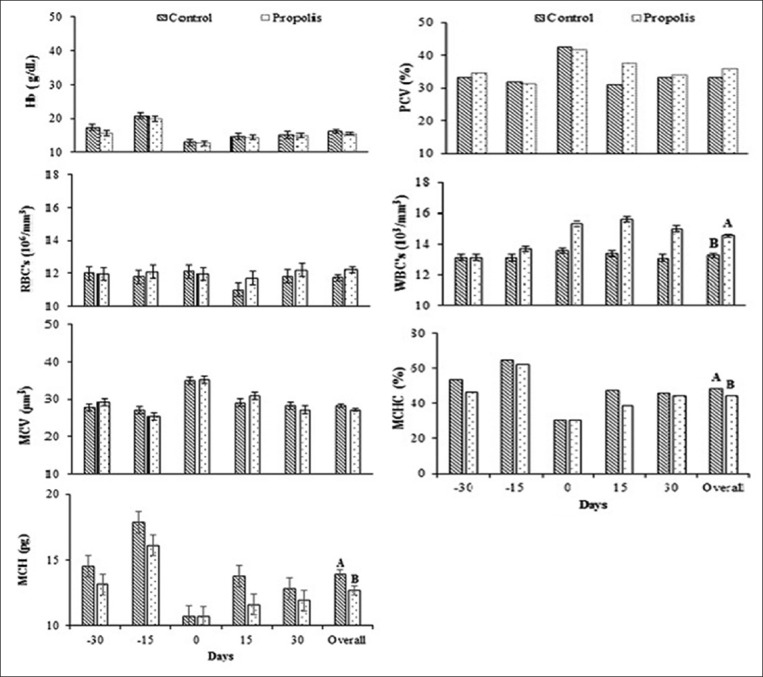
Changes of some hematological parameters in Barki ewes fed a basic diet or a diet supplemented with propolis during late pregnancy and early lactation under arid conditions. ^AB^Letters among the group differ significantly (p<0.05). Days (−30 and −15) = days of late pregnancy, day 0 = day of parturition, days (15 and 30) = days of early lactation.

Data for hematological parameters in Barki ewes are shown in Figure-3. Overall means of blood Hb concentration (g/dl), PCV, %, and MCV, µm^3^ in the propolis group were not different from the control. Numerical increment in overall means of RBC counts (10^6^/mm^3^) was found in propolis group (12.22±0.18 10^6^/mm^3^) compared with control (12.22±0.18 10^6^/mm^3^) with no significant difference. Overall means of (RBCs, 10^6^/mm^3^) were not affected by time (−30, −15, 0, 15, and 30 days) with no treatment × time interaction. Overall means of leukocyte counts (WBC’s, 10^3^/mm^3^) were significantly increased (p<0.05) by treatment (14.52±0.08 10^3^/mm^3^) compared to control (13.24±0.08 10^3^/mm^3^). There was an interaction (p<0.05) between treatment and time (−30, −15, 0, 15, and 30). In the treatment group, the WBC’s 10^3^/mm^3^ increased in days 0, 15, and 30 (15.27±0.19, 15.61±0.19, and 14.96±0.19, respectively), concurrent with a decrease in the control group in the respective days (13.56±0.20, 13.37±0.20, and 13.07±0.20 10^3^/mm^3^, respectively). A significant (p<0.05) decline in overall means of mean corpuscular Hb (MCH, pg) was found in the treatment group (12.68±0.35 pg) compared with control (13.91±0.36 pg). There was no treatment × time interaction. The same trend was found in the overall of corpuscular Hb concentration (MCHC, %) where significant percent decreased in the treatment group (44.31 %) compared with control (48.41%) with no treatment × time interaction.

### Changes in milk yield and milk composition

The changes in milk yield and milk composition of Barki ewes fed diet supplemented with Chinese propolis are shown in Table-5. The overall mean of milk yield (mL/day) significantly (p<0.05) increased in propolis group (159.50 ml/day) compared to control (130.00 mL/day). Milk yield declined with the advancement of lactation; highest milk yields were recorded at weeks 4 and 6 (170, 284.0 mL/day, respectively), while the lowest was recorded at week 8 (108 mL/ ay). No treatment × time interaction (p=0.18) was observed on weekly milk yields. No treatment × time interaction was observed on milk protein, fat, lactose, TSs, SNF, and ash content. Overall means of milk protein, lactose, SNF, and ash were not different between propolis and control groups Table-5. However, overall mean of milk fat (g/100 mL) significantly (p<0.01) increased in propolis group (6.23 g/100 mL) compared to control (4.59 g/100 mL). Overall mean of milk TSs (g/100 mL) significantly (p<0.01) increased in propolis group (15.86 g/100 mL) compared to control group (13.95 g/100 mL).

**Table-5:** Changes in MY and milk composition of Barki ewes in groups during early lactation period.

Items	Treat.	Weeks				Overall	SEM	p-value		
	
		2	4	6	8			T	W	T×W
MY, g/day	Control	90.0	87.5	220.0	122.5	130^B^	22.5	0.05	0.01	0.18
	Propolis	112.0	170.0	284.0	108.0	168.5^A^	22.5			
Protein, g/100 mL	Control	3.42	3.51	3.43	3.58	3.48	0.12	0.19	0.38	0.94
	Propolis	3.49	3.71	3.52	3.68	3.60	0.11			
Fat, (g/100 mL)	Control	3.80	4.64	4.62	5.30	4.59^B^	0.63	0.01	0.01	0.45
	Propolis	4.66	6.05	6.14	8.09	6.23^A^	0.56			
Lactose, (g/100 mL)	Control	5.15	5.21	5.16	5.38	5.22	0.23	0.41	0.33	0.75
	Propolis	5.02	5.55	5.30	5.55	5.35	0.20			
Ash, (g/100 mL)	Control	0.76	0.77	0.76	0.80	0.77	0.03	0.11	0.40	0.85
	Propolis	0.77	0.83	0.80	0.83	0.80	0.02			
TS, (g/100 mL)	Control	12.58	14.12	14.01	15.10	13.95^B^	0.88	0.01	0.01	0.51
	Propolis	13.12	16.33	15.78	18.19	15.86^A^	0.79			
SNF, (g/100 mL)	Control	9.38	9.48	9.39	9.80	9.51	0.38	0.18	0.39	0.79
	Propolis	9.46	10.27	9.64	10.10	9.87	0.34			

Days 15 and 30: Days of early lactation. Days −30 and −15: Days of late pregnancy, Day 0: Day of parturition. MY=Milk yield, TS=Total solid, SNF=Solid not fat

### Newly born lambs

#### Body weight of lambs

**Figure-4:**
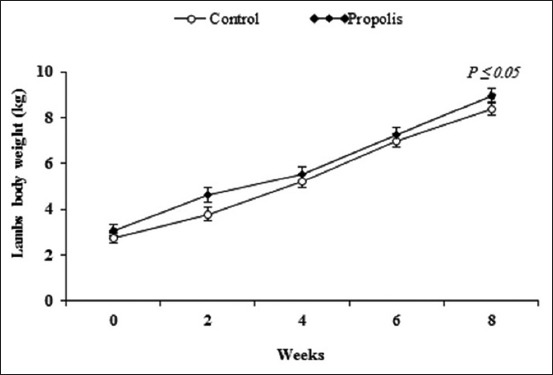
Changes of newly born lambs’ body weights (kg) born from Barki ewes fed a basic diet or supplemented with propolis during early lactation period under arid condition. Week 0 = birth weight. Weeks 2-8 = weekly body weights from parturition to weaning (week 8).

Data for newly born lambs body weight from birth to weaning (8 weeks) are shown in Figure-4. Weaning weight for lambs born from ewes supplemented with Chinese propolis significantly (p<0.05) increased (8.96±0.32 kg) compared with lambs born from control ewes (8.36±0.27 kg). No treatment × time interaction (p=0.74) was observed on lambs body weights.

#### Thermo-cardiorespiratory responses of lambs

Mean values of thermo-cardiorespiratory responses including rectal, skin, and CTs, heart, and RRs of lambs are shown in Table-6 during early weaning period (60 days after parturition). The results indicated that propolis supplementation to ewes had no significant effect on lambs overall RT (RT, 39.02±1.30°C), ST (ST, 36.32±0.17°C), RR (RR 49.09±0.33 rpm), and HR (HR, 126.09±1.76 bpm) compared with control lambs (RT, 42.19±1.64°C; ST, 36.00±0.21°C; RR, 47.65±0.90 rpm; and HR, 128.25±2.22 bpm), respectively. However, CT was higher (p<0.05) in propolis lambs (35.45±0.29°C) compared with control lambs (35.45±0.29°C).

**Table-6:** Changes in some thermo-cardiorespiratory responses of groups under arid conditions (mean±SE).

Items	Overall treat.		Overall hours		Overall days	
		
	Control	Propolis	8:00 AM	2:00 PM	30	60
RT (°C)	42.19±1.64	39.02±1.30	41.74±1.48	39.47±1.48	39.14±1.48	42.07±1.48
ST (°C)	36.00±0.21	36.32±0.17	35.88±0.19	36.44±0.19	36.46±0.19	35.86±0.19
CT (°C)	33.81±0.37^B^	35.45±0.29^A^	33.60±0.33	35.66±0.33	34.62±0.33	34.64±0.33
RR (rpm)	47.65±0.90	49.09±0.33	45.21±0.81	51.52±0.81	48.81±0.81	47.93±0.81
HR (bpm)	128.25±2.22	126.09±1.76	124.03±2.0	130.30±2.0	128.66±2.00	125.67±2.0

A,B Values within the same row with different letters differ significantly (p<0.05). Propolis lambs: Lambs born for ewes supplemented with propolis; control lambs: Lambs born for control ewes. RT=Rectal temperature, ST=Skin temperature, CT=Coat temperature, RR=Respiration rate, HR=Heart rate, SE=Standard error

#### Changes of some antioxidant activities in lambs

**Figure-5:**
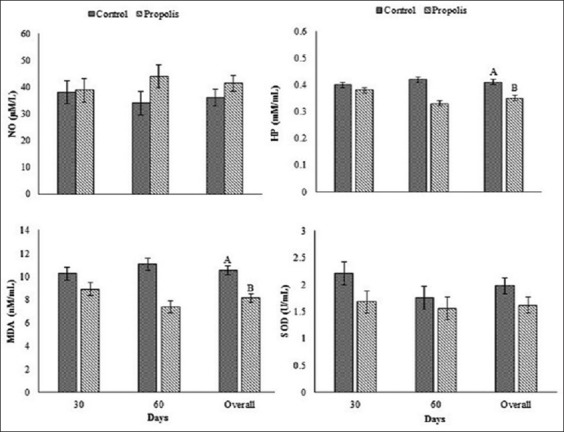
Changes of some plasma antioxidant enzyme activities in lambs born for Barki ewes fed a basic diet or supplemented with propolis during early lactation period under arid conditions. ^AB^Letters among the group differ significantly (p<0.01).

Changes in plasma antioxidant activity’s concentrations in Barki lambs are shown in Figure-5. No treatment × time interaction was detected on overall mean concentrations of NO (µM/L) and SOD (U/ mL). No significant effect was detected in overall mean concentrations of NO (µM/L) and SOD (U/mL) in propolis lambs (41.46±3.06 µM/L and 1.62±0.15 U/mL) compared with control (36.11±3.06 µM/L and 1.98±0.15 U/mL), respectively. No treatment × time interaction was detected on overall mean concentrations of HP (mM/mL) and MDA (nM/mL). Overall mean concentrations of MDA and HP were significantly (p<0.01) decreased in propolis lambs compared with control lambs (MDA, 8.15±0.38 vs. 10.56±0.38 nM/mL and HP, 0.35±0.01 vs. 0.41±0.01 mM/mL), respectively.

## Discussion

Values of (IgA) were significantly increased in propolis group compared with control, also (IgM) values tended to increase significantly in propolis group as well, while no significance was detected in IgG values in propolis group compared with control. It is well known that increased immunity activates defensive mechanisms of animals against microbial diseases that may attack animals’ pre and after parturition under arid conditions [21]. Several factors such as nutrition, dry weather, and health condition can have negative effects on Igs (A, G, M) by decreasing the cell-mediated immune response, which led to degradation efficiency of the thymus, lymph nodes, and spleen in sheep and goats [4,21]. These results were in agreement with those reported by Emtnan *et al*. [22] who found that Ig concentration improved by supplementation of propolis to Baladi goats. These results might be attributed to unknown compounds and biologically active compounds such as phenol compounds and flavonoids, which positively affect the humoral immune response [23].

Overall means of SOD, HP, and NO concentrations, activity were significantly decreased in propolis group compared with control, while the overall mean of MDA concentrations activity tended to significantly decrease in propolis group compared with control. Reactive nitrogen species and reactive oxygen species (ROS) are produced as results of many internal and external pathways as well as cellular metabolites or as a response to climate and toxicity of the surrounding environment. This has a negative effect on human and animal health [24]. Propolis would have effects on antioxidant activity depending on its chemical composition, which varies among different propolis types [25]. Phenolic acids and flavonoids are characterized by their powerful antioxidant activity. They inhibit the activity of some enzymes (protein kinase C, xanthine oxidase, cyclooxygenase, ascorbic acid oxidase, lipoxygenase, ATPase, and cAMP phosphodiesterase) which inhibit the production of ROS species by interrupting the reactions leading to the lipid peroxidation that is involved in the process of free radical creation [26].

No significant differences were detected in overall means of plasma T_3_ and (T_4_) concentrations between treatment and control groups. Kandiel *et al*. [1] and Doaa *et al*. [27] reported that serum T_3_ and T_4_ concentration were not different among different stages of pregnancy and postpartum period in sheep. The same results were found in dairy cows during the pre-calving and post-calving period [28]. These results may be attributed to alterations in cardiac output and increased blood volume [29,30]. Organification is a biochemical process that takes place in thyroid glands. It is the incorporation of iodine into thyroglobulin for the production of T_4_ and T_3_, a step done after the oxidation of iodide by the enzyme thyroid peroxidase in the presence of HP, generated by thyroid dual oxidases (DuOx1 and 2) [31]. Flavonoids are potent natural plant-derived compounds, which are capable of interference with thyroid hormone economy [32]. In addition, flavonoids could exert goitrogenic effects *in vivo* and inhibit the organification of iodide *in vitro*; however, the mechanism by which flavonoids block thyroid hormone synthesis was not known yet [33]. Some studies explained the inhibitory effects of flavonoids on thyroid hormones secretion. Divi and Doerge [34] concluded that inhibition of thyroid hormones secretion starts earlier during the iodination processes of the amino acid tyrosine in the thyroid gland.

Provision of Chinese propolis supplement before parturition and during lactation period increased overall means of WBCs (10^3^/mm^3^) at 0, 15, and 30 days in supplemented ewes, while the control group showed a decrease in the respective days. Overall means of RBCs counts (10^6^/mm^3^) tended to increase in the treated group compared with control. The present results showed that Hb, g/dl, MCV, µm^3^ concentrations, and PCV, % were not different among the treatment and control group. In agreement with our results, Morsy *et al*. [35] concluded that using extracted red Brazilian propolis (3 g/h/days) to 21 days in Santa Ines ewes before lambing enhanced total leukocyte count while Hb and PCV were not affected. The total WBCs and RBCs were increased while MCV was decreased when New Zealand white bucks fed on 150 mg/kg propolis [36]. In Hanwoo calves, the propolis powder (0.05%) was supplemented with concentrate diets slightly increased WBCs [5].

Propolis is one of the natural substances which have positive immunomodulatory effects on human and animals. In recent years, studies have provided more information about the effect of propolis on the immune system [12]. In the present study, the increase of WBCs may be due to the role of propolis (flavonoids) in the upregulating of expression of toll-like receptors (TLR-2, TLR-4) and enhancing the production of cytokines (interleukin [IL]-1, IL-6, and IL-10) by spleen cells [12]. Moreover, propolis is considered an antioxidant that can stimulate the antioxidants enzymes such as MDA, HP, SOD, NO to reduce the free radicals, leading to the preservation, and enhancing function efficiency of immune organs [37,38]. These results are supported by the data presented in Table-4. Our results showed that plasma IgA concentration was significantly increased in ewes fed on a diet supplemented with propolis compared with control. The same trend was found in IgM concentration, but this increase was not significant while IgG concentration was not different between the two groups.

In Egypt, sheep milk is a secondary product to meat but is of high importance in coastal regions and oasis’s [3]. Morsy *et al*. [35] reported that administration of Brazilian red propolis extracts to Santa Ines ewes (3 g/h/days) up to the 21^st^ day after parturition increased milk yield, fat, protein and lactose yield, and energy corrected milk. In Baladi goats, the same results were found by Emtnan *et al*. [22]. In dairy cows, the addition of propolis enhanced milk yield and quality of milk fat composition and antioxidant capacity of milk [39]. In the current results, the improvement in milk yield, milk fat could be attributed to improved oxidative stress conditions displayed in the form of decreased antioxidant enzymatic activities resulting in enhanced productivity of ewes [40].

The present results revealed that body weight of lambs born from ewes fed a diet supplemented with Chinese propolis was heavier at weaning (week 8) than controls. Similar results were previously reported by Sarker and Yang [5] and Emtnan *et al*. [22]. Variation of milk production is a characteristic of arid regions. Therefore, lamb’s growth is associated directly with enhanced milk production during the early lactation period, first 30 days after birth [41]. Later on lamb’s life, the lamb gradually moves away from full reliance on milk to conventional diets [42]. In addition, flavonoid compounds present in propolis can help improve gut health and attenuate diarrhea in young calves and can improve growth in young calves [23]. It is known that the milk composition has a positive or negative effect by fed supplementation, such as propolis or drugs such as antibiotics throughout lactation period and this, therefore, has an impact on the health status of calves [23]. These data are associated with decreased overall mean MDA and pH concentrations in lambs born to ewes fed Chinese propolis supplemented diets. Antioxidants in the propolis such as phenolic acids and flavonoids are characterized by their powerful antioxidant activity [23] and are known to inhibit the production of ROS species and hence preserve lipids from peroxidation [26].

The present study showed no significant differences among lambs ewes fed Chinese propolis supplemented diets and control groups in overall mean values of thermo-cardiorespiratory responses (RT, ST, RR, and HR), while CT value was higher in treated lambs than control. In general, thermo-cardiorespiratory responses were affected by several factors such as environmental condition, feeding, breed, and sex [43,44]. RT is generally used as a measurement of animal core temperature [45]. Furthermore, it is well known that ST and CT are regulated by blood flow to the skin and both evaporation and radiation from the skin [46]. RR and HR have been used as an accurate tool to measure the severity of thermal stress on an animal and reflect the homeostasis of circulation and the general metabolic status. These values of thermo-cardiorespiratory responses including (RT, ST, CT, RR, and HR) are within the reference values during the weaning period in Barki lambs under arid conditions in Siwa Oasis [47].

## Conclusion

It can be concluded that Chinese propolis supplementation to peri-parturient ewes improved the concentrations of the antioxidant enzymes including SOD, HP concentrations, NO concentrations, and MDA and enhanced functions of the immune system, and improved milk production and weaning weight, antioxidant status of born lambs under arid conditions. However, thermo-cardiorespiratory responses of lambs under arid condition were not altered by Chinese propolis supplementation.

## Authors’ Contributions

ISA helped in carrying out blood and plasma biochemical analysis in DRC complex laboratories and writing this article. EAE helped with providing some nutritional materials and analysis of the chemical composition of diets and milk samples in laboratories of faculty of agriculture, Alexandria University. HAS, BF, and MA-HE helped to collect blood samples and field data during the experiment period. All authors helped in planning the research and statistical analysis of the results and all contributed in drafting the manuscript. All authors read and approved the final manuscript.
